# Role of DSM5 Anxious Distress Specifier Interview in symptoms severity and medication adherence in 1st episode mania

**DOI:** 10.1192/j.eurpsy.2022.1015

**Published:** 2022-09-01

**Authors:** S. El Sayed, S. Gomaa, Z. Gomaa

**Affiliations:** Faculty of Medicine-Mansoura University, Department Of Psychiatry, faculty Of Medicine, Mansoura University, Mansoura, Egypt

**Keywords:** severity, Anxious, mania, Adherence

## Abstract

**Introduction:**

-Anxious Distress Specifier is one of the newly added specifier in diagnosis and managment of bipolar disorder.This unique item may paly a role in not only the symptoms severity but also the degree of adherence to the psychotropics. -DSM5 Anxious distress specifier is not well studied in the 1st manic episode of bipolar disorder.

**Objectives:**

1-To study the role of DSM5 Anxious Distress Specifier in the symptoms severity of 1st diagnosed manic episode 2-To investigate its role in medication adherence in thses patients

**Methods:**

1-DSM 5 Anxious distress specifier interview which includes 5 items : a- Keyed up or tense b-Restlessness c-Impaired concentration. d-Sense of foreboding e-Loss of control 2-The Young Mania Rating Scale (YMRS) is one of the most frequently utilized rating scales to assess manic symptoms. The scale has 11 items and is based on the patient’s subjective report of his or her clinical condition 3-Drug Attitude Inventory:consists of a questionnaire that is completed by the patient, pertaining to various aspects of the patient’s perceptions and experiences of treatment.

**Results:**

1-There is a positive correlation between the mean score of Young mania Rating scale in 1st episode manic patients and the mean score of DSM5 Anxious Distress specifier Interview 2-The presence of high score of DSM5 Anxious Distress Specifier Interview is positively correlated to the mean score of Drug Attitude Inventory during the follow up visits after controlling the 1st episode mania

**Conclusions:**

The presence of high levels of Anxious Distress in the 1st episde mania affected the symptoms severity and medication adherence

**Disclosure:**

No significant relationships.

